# Hair Cortisol and Health-Related Quality of Life in Children with Mental Disorder

**DOI:** 10.1177/24705470211047885

**Published:** 2021-10-12

**Authors:** M. Claire Buchan, Sydney Whitney, Scott T. Leatherdale, John G. Mielke, Andrea Gonzalez, Mark A. Ferro

**Affiliations:** 1 8430 School of Public Health Sciences, University of Waterloo, Waterloo, ON, Canada; 2 3710 Department of Psychiatry and Behavioural Neurosciences, McMaster University, Hamilton, ON, Canada

**Keywords:** HPA-axis, hair cortisol, children, quality of Life, psychopathology, comorbidity

## Abstract

**Introduction:**

Children living with mental disorder are at risk for lower health-related quality of life (HRQoL) compared to their peers. While evidence suggests that cortisol dysregulation is implicated in the onset of mental disorder, the extent to which cortisol is associated with HRQoL is largely unknown. Further, it remains unknown how comorbid physical illness may alter this relationship. This study examined whether the presence of a comorbid physical illness moderated the association between hair cortisol concentration (HCC) and HRQoL among children with mental disorder.

**Methods:**

One-hundred children (4-17 years) receiving care from a pediatric hospital were recruited. The Mini International Neuropsychiatric Interview was used to measure mental disorder and the KIDSCREEN-27 to assess HRQoL. Cortisol extracted from children's hair was assayed using high-sensitivity ELISA. Multiple regression analyses tested the association between HCC and HRQoL.

**Results:**

Presence of a physical illness was found to moderate the relationship between HCC and HRQoL in the domain of peers and social support [comorbidity: β = −0.57 (−0.97, −0.17); no comorbidity: β = 0.22 (−0.11, 0.55)].

**Conclusion:**

The association between HCC and HRQoL in children with mental disorder is moderated by the presence of a physical illness, such that in children with comorbid physical and mental disorder, elevated HCC is associated with lower HRQoL. Approaches that reduce stress in these children may help promote optimal well-being. More research investigating physiological stress and psychosocial outcomes in children with mental disorder, particularly those with comorbid physical illness, is needed.

## Introduction

The burden associated with childhood mental disorder is considerable and may contribute to increased levels of perceived^1^ and physiological stress^2^ in these children and adolescents. Physical and mental disorder are strongly associated^3–5^ and evidence suggests that comorbid physical and mental disorder negatively affects multiple domains of child and adolescent health-related quality of life (HRQoL), beyond the effect of having either type of condition in isolation.^6,7^

The hypothalamic-pituitary adrenal (HPA) axis is believed to play a critical role in the pathophysiology of mental disorders. HPA dysregulation can be the result of prolonged exposure to stress or traumatic events,^2,8^ and studies show that children and adolescents with mental disorder exhibit altered HPA axis function.^9,10^ This is also evident in the context of physical illness whereby HPA dysregulation, as measured by hair cortisol concentration (HCC), was found to be associated with the onset of mental disorder in children and adolescents with physical illness.^11^

The extent to which cortisol is associated with HRQoL in children and adolescents is unclear. One study found no association between HCC and HRQoL among healthy children,^12^ and the second found a positive association between HCC and HRQoL only among high-risk children whose mothers had been exposed to early-life maltreatment (ie, moderating effect).^13^ In the latter study, it was suggested that HPA axis dysregulation among high-risk children was attributable to experiencing higher levels of chronic stress.^8^

Given the robust evidence that physical and mental disorders are often comorbid and emerging reports that both are associated with elevated stress and compromises to HRQoL, we investigated whether the association between HCC and HRQoL in children and adolescents with mental disorder was moderated by the presence of a physical illness. We hypothesized that physical illness would moderate the association such that elevated HCC would be associated with lower HRQoL in children and adolescents with comorbid physical and mental disorder compared to those with mental disorder only.

## Methods

Children and adolescents (4-17 years) and their families were recruited from physical and mental outpatient clinics at two pediatric hospitals in Ontario, Canada; the details of recruitment and study design are described in detail elsewhere.^14^ A total of 321 families were identified, 150 (47%) of which completed diagnostic telephone interviews. Of the 114 (76%) children and adolescents who screened positive for mental disorder, 100 provided complete data and were included in our analyses. There were no sociodemographic, or health-related differences between participants and non-participants. Ethical approval was obtained from the Hamilton Integrated Research Ethics Board (15-197; 14-130) and Western Research Ethics Board (105 505) prior to conducting the study. All procedures were in accordance with the ethical standards of the institutional guidelines and with the 1964 Helsinki declaration.

Screening for mental disorder occurred via telephone using parent responses to the Mini International Neuropsychiatric Interview for Children and Adolescents (MINI-KID), a brief diagnostic interview validated for assessing DSM-IV disorders in individuals ≤17 years of age.^15^ Youth, aged 8 to 17, were asked to complete the interview themselves, and parents of youth <8 completed the proxy version on their behalf. The MINI-KID has demonstrated robust psychometric properties^16,17^ and good concordance between the child and parent versions.^15^ The timeframe of symptom assessment was six months.

HRQoL across the domains of physical well-being, psychological well-being, autonomy and parent relations, social support and peers, and school environment was measured using the KIDSCREEN-27.^18^ The KIDSCREEN-27 has been validated for children and adolescents with and without physical and mental disorders and has good parent-child agreement.^19–21^ Raw scores are transformed to T scores with mean 50 and standard deviation 10. Parent reports were used as 19 children were age-ineligible to complete the KIDSCREEN-27.

Cortisol was extracted from child and adolescent hair samples during in-person data collection (pre-COVID). Approximately 50 to 60 dry hairs were collected from the posterior vertex of the head, attached to a sheet of paper with a paper clip, and marked to indicate direction of growth. Hair samples were accompanied by a questionnaire completed by parents that detailed variables hypothesized to affect HCC [eg, medication use, hair washing and treatments, smoke exposure, etc.]^22^ The protocol for hair processing was based on that of Vaghri et al^23^ and has been previously used successfully.^11,24^ Samples were analyzed using enzyme-linked immunosorbent assay (ELISA) using the High Sensitivity Salivary Cortisol Immunoassay Kit (Cat# 1-3002, Salimetrics, Pennsylvania).

Presence of a comorbid physical illness and corticosteroid medication use were collected using standardized questions and completed by parents. Sociodemographic information was also collected via a standardized form and included child and parent age, sex, and immigration status, parent education and marital status, and household income.

Univariable statistics were used to describe the sociodemographic and health-related characteristics of the sample. Multiple regression was conducted to examine the association between HCC and parent-reported HRQoL. These models were stratified by the physical illness status and adjusted for the potential confounding effects of child age, sex, and use of corticosteroids.^22^ Standardized coefficients with 95% confidence internals were reported. In the presence of a significant main effect of HCC in either stratified model (mental disorder only, *n* = 53 or comorbid physical and mental disorder, *n* = 47), moderating effects were investigated. These post hoc regression models were computed using derived conditional moderator variables centered on zero, which allowed for the examination of two-way interaction effects of physical illness status on the relationship between HCC and HRQoL.^25^ Analyses were conducted using SAS 9.4.

## Results

**Figure 1.**
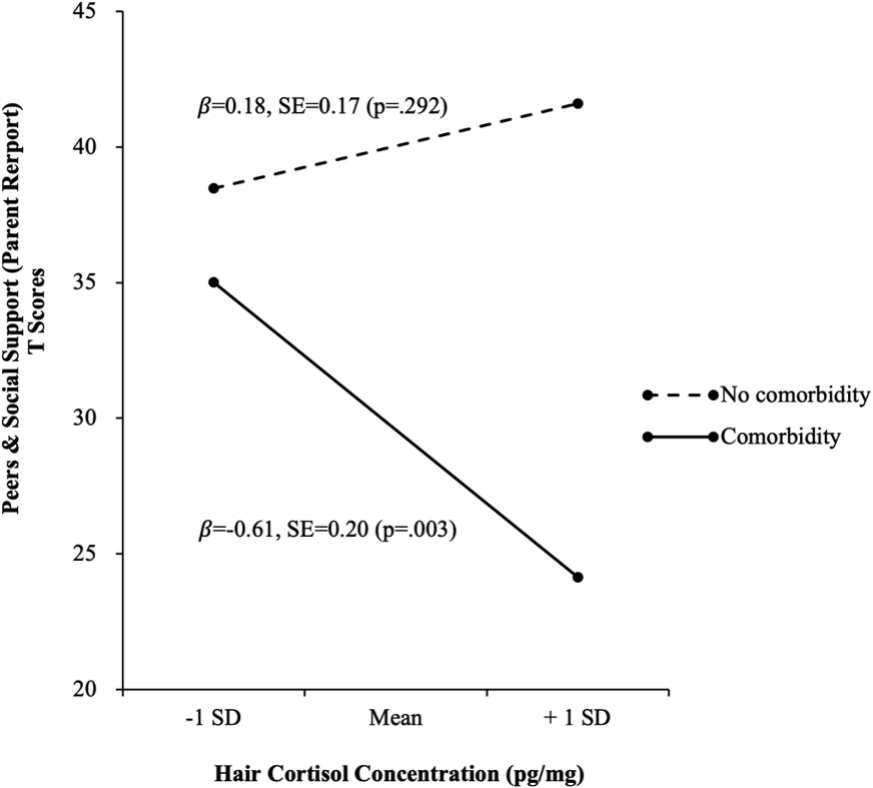
Effect modification of having a comorbid physical illness on the association between hair cortisol concentration and the domain of peers and social support.

Descriptive statistics are reported in Table 1. Significant differences between the two samples were found with child age, hair colour, and corticosteroid use. Children with comorbid physical and mental disorders were reported to be younger in age and reported use of corticosteroids for the management of their physical disorders. Additionally, significant differences were identified in parental marital status, household income, and parental stress scores. Table 2 presents the associations between HCC and HRQoL stratified by physical illness status. Among children and adolescents with mental disorder only, HCC was not associated with HRQoL in any domain (in both unadjusted and adjusted models). Among children and adolescents with comorbid physical and mental disorder, higher HCC was associated with lower HRQoL scores in the domain of peers and social support [β = −0.57, 95% CI: −0.97, −0.17] (Table 2). This moderating effect is illustrated in Figure 1.

**Table 1.** Sample characteristics.

Characteristic	Mental Disorder Frequency (%)	Mental & Physical Disorder Frequency (%)	*P*-value
Child			
Age (years, mean [sd])	**13.89 (3.0)**	**12.04 (3.7)**	**.007**
Female	37 (70)	30 (64)	.5255
Hair colour			
Blonde	**15 (29)**	**10 (22)**	**.0320**
Brown	**31 (60)**	**34 (74)**	
Black	**0 (0)**	**2 (4)**	
Red	**6 (11)**	**0 (0)**	
Hair washing (# per week, mean [sd])	3.69 (1.6)	3.95 (0.5)	.5025
Smoke exposure (*n* exposed, mean [sd])	16 (32)	13 (29)	.7423
Hair cortisol concentration (pg/mg, mean [sd])	11.89 (9.3)	10.09 (8.4)	.3114
Health-related Quality of Life (mean [sd])			
Physical Well-being	**37.20 (8.3)**	**42.92 (10.9)**	**.0036**
Psychological Well-being	**29.58 (3.2)**	**38.51 (9.7)**	**<.0001**
Parents & Autonomy	42.44 (7.9)	45.00 (7.7)	.1089
Peers & Social Support	38.56 (11.8)	42.28 (11.4)	.1137
School Environment	**38.83 (11.3)**	**43.67 (10.0)**	**.0296**
Mental Disorder			
Internalizing disorder	27 (51)	25 (53)	.9610
Externalizing disorder	4 (8)	3 (6)	
Comorbid internalizing & externalizing disorders	22(41)	19 (40)	
Corticosteroid medication use	**0**	**9 (19)**	**.0008**
Parent			
Income ≥$75,000	**25 (47)**	**33 (73)**	**.0086**
Completed college/university	34 (64)	32 (68)	.2546
Marital status (partnered)	**30 (57)**	**36 (77)**	**.0352**
Depression (CES-D, mean [sd])	21.07 (10.4)	17.20 (10.6)	.0732
Anxiety (STAI, mean [sd])	43.32 (7.9)	45.30 (9.0)	.244
Parental Stress (PSS, mean [sd])	**51.24 (12.5)**	**44.0 (12.9)**	**.005**

Data are presented as *n* unless otherwise specified. Sample *n* = 100 and therefore frequency denotes both *n* and percentage unless otherwise specified. Statistically significant coefficients (*p* ≤ 0.05) are shown in bold.

**Table 2.** Association between hair cortisol concentrations and parent-reported health-related quality of life among children with mental disorder and those with comorbid mental and physical disorders.

Health Related Quality of Life	Mental Disorder (*n* = 53)		Comorbid Physical & Mental Disorder (*n* = 47)	
	Unadjusted	Adjusted	Unadjusted	Adjusted
Physical Well-being	0.24 (−0.00, 0.48) *P* = .056	0.14 (−0.07, 0.35) *P* = .181	0.21 (−0.17, 0.59) *P* = .284	0.02 (−0.34, 0.37) *P* = .920
Psychological Well-being	−0.02 (−0.11, 0.08) *P* = .739	−0.05 (−0.14, 0.04) *P* = .264	−0.05 (−0.40, 0.29) *P* = .768	−0.17 (−0.51, 0.18) *P* = .334
Parents & Autonomy	0.04 (−0.20, 0.28) *P* = .715	−0.01 (−0.25, 0.23) *P* = .944	−0.02 (−0.32, 0.28) *P* = .892	−0.04 (−0.35, 0.28) *P* = .862
Peers & Social Support	0.18 (−0.17, 0.54) *P* = .306	0.22 (−0.11, 0.55) *P* = .180	**−0.49 (−0.87,−0.11)** *P* = .013	**−0.57 (−0.97,−0.17)** *P* = .007
School Environment	−0.08 (−0.43, 0.26) *P* = .624	−0.17 (−0.50, 0.16) *P* = .312	−0.16 (−0.52, 0.19) *P* = .364	−0.33 (−0.68, 0.00) *P* = .049

Data are standardized coefficients (95% confidence intervals). Adjusted models accounted for the potential confounding effects of child age, sex, and use of corticosteroids. Statistically significant coefficients (*p* < 0.05) are shown in bold.

## Discussion

This study showed that higher HCC was associated with lower HRQoL in the domain of peers and social support, but only among children and adolescents with a comorbid physical illness. This moderating effect is consistent with previous research that found children and adolescents with comorbid physical and mental disorder report more impairments in their HRQoL compared to those with either consider independently^6,7^ and may be attributable to the added burden associated with having a comorbid physical illness, particularly in the context of friendships. Children and adolescents with more severe physical impairments report having difficulty building or maintaining social relationships with peers^26^ and have been shown to be more likely to be victims, or perpetrators, of bullying behaviors.^27^ Notably, bullying during childhood and adolescence has been linked with impaired mental health,^28^ increased stress,^29^ and altered cortisol responses.^30^ Cumulatively, these effects may explain the association between HCC and lower HRQoL in our sample of high-risk children and adolescents. We encourage additional research with larger samples to replicate or refute our findings, as well as to expand the field by examining more nuanced associations of HCC and HRQoL in children with physical disorder independently, and specific physical-mental comorbidities.

There are limitations to our work that warrant consideration. The cross- nature of the data did not allow for temporal associations to be examined and relevant clinical characteristics (ie, symptom severity and time since onset of symptoms) could not be measured.^31^ Additionally, the relatively small sample size limited our ability to draw comparisons between specific physical-mental comorbidities. Given the substantial burden that families struggling with children with severe physical and/or mental disorders experience, it is possible that self-selection bias influenced our final study sample. While the MINI-KID has been previously validated in youth populations,^15^ self-report measures – particularly when administered to youth – are subject to recall bias.^32^ As such, these findings should be replicated using other informants or data sources (eg, physician-report, medical charts). Similarly, due to limitations in our study sample, parent reports of HRQoL were used. This may introduce reporting biases and ultimately influence the relationships identified in this study.^14^ Finally, a control group was not included in the study population rendering it impossible to test whether HCC in children with mental disorder differed significantly from HCC of healthy children; however, it is conceivable that the relationships found in this study would also be found among children without mental disorder.

In this study, having a comorbid physical illness moderated the association between elevated HCC and lower HRQoL among children and adolescents with mental disorder. Opportunities to reduce stress among children and adolescents with comorbid physical and mental disorder, perhaps in school settings, should be explored to support these high-risk individuals and promote optimal HRQoL. Future research should advance the research agenda by exploring associations of chronic stress and HPA dysfunction to contextualize the findings of this study and to gain a better understanding of how physical-mental comorbidity influences HRQoL in children and adolescents.
